# Maximising the wealth of few at the expense of the health of many: a public health analysis of market power and corporate wealth and income distribution in the global soft drink market

**DOI:** 10.1186/s12992-021-00781-6

**Published:** 2021-12-02

**Authors:** Benjamin Wood, Phil Baker, Gyorgy Scrinis, David McCoy, Owain Williams, Gary Sacks

**Affiliations:** 1 Global Obesity Centre, Deakin University, Geelong, Australia; 2 Institute for Physical Activity and Nutrition, Deakin University, Geelong, Australia; 3 School of Agriculture and Food, University of Melbourne, Melbourne, Australia; 4 Institute of Population Health Sciences, Queen Mary University London, London, UK; 5 School of Political Science and International Studies, University of Leeds, Leeds, UK

**Keywords:** Global soft drink industry, Market power, Wealth and income distribution, Distributive injustice, Sustainable development

## Abstract

**Background:**

Many of the harms created by the global soft drink industry that directly influence human and planetary health are well documented. However, some of the ways in which the industry indirectly affects population health, via various socio-economic pathways, have received less attention. This paper aimed to analyse the extent to which market power and corporate wealth and income distribution in the global soft drink market negatively impact public health and health equity. In doing so, the paper sought to contribute to the development of a broad-based public health approach to market analysis. A range of dimensions (e.g., market concentration; financial performance; corporate wealth and income distribution) and indicators (e.g., Herfindahl Hirschman Index; earnings relative to the industry average; effective tax rates; and shareholder value ratios) were descriptively analysed. Empirical focus was placed on the two dominant global soft drink manufacturers.

**Results:**

Coca-Cola Co, and, to a lesser extent, PepsiCo, operate across an extensive patchwork of highly concentrated markets. Both corporations control vast amounts of wealth and resources, and are able to allocate relatively large amounts of money to potentially harmful practices, such as extensive marketing of unhealthy products. Over recent decades, the proportion of wealth and income transferred by these firms to their shareholders has increased substantially; whereas the proportion of wealth and income redistributed by these two firms to the public via income taxes has considerably decreased. Meanwhile, the distribution of soft drink consumption is becoming increasingly skewed towards population groups in low and middle-income countries (LMICs).

**Conclusions:**

Market power and corporate wealth and income distribution in the global soft drink market likely compound the market’s maldistribution of harms, and indirectly influence health by contributing to social and economic inequalities. Indeed, a ‘double burden of maldistribution’ pattern can be seen, wherein the wealth of the shareholders of the market’s dominant corporations, a group over-represented by a small and wealthy elite, is maximised largely at the expense of the welfare of LMICs and lower socioeconomic groups in high-income countries. If this pattern continues, the appropriate role of the global soft drink market as part of sustainable economic development will require rethinking.

**Supplementary Information:**

The online version contains supplementary material available at 10.1186/s12992-021-00781-6.

## Introduction

As with a number of other unhealthy commodity industries, many of the health and ecological harms created by the global soft drink industry are well recognised, such as those related to added sugars and plastic pollution [1–9]. As an example, the consumption of sugar-sweetened beverages (SSBs) – the core product of the industry – is positively associated with a higher risk of death from all causes [10, 11]. In 2010 alone, SSB consumption contributed to an estimated 184,000 deaths and 8.5 million disability-adjusted life years worldwide [10, 11]. Additionally, the global soft drink industry is a major contributor to plastic pollution entering terrestrial and marine ecosystems [8, 12, 13]. The two largest global soft drink manufacturers by market share – Coca-Cola Co and PepsiCo – are also the world’s two largest manufacturers of plastic packaging [8, 14]. Combined, these two firms produce at least 5.3 million tonnes of plastic packaging every year, of which an estimated 337,000 tonnes ends up polluting ecosystems around the world [8, 14].

Many of the harms associated with the global soft drink market are distributed in a manner that impacts health and social equity. For instance, low and middle-income countries (LMICs) are disproportionately burdened with the deaths and disabilities linked to SSB consumption [11]. The health-related burden of SSB consumption in LMICs is likely to be dynamic across social groups, in which consumption after market entry tends to increase first among higher-income groups before shifting to lower-income groups as countries become wealthier [15]. LMICs are also more likely to be burdened by the harms created by plastic pollution, in part because they are more likely to lack the required waste management capacity to deal with vast amounts of plastic waste [16]. International trade in plastic waste exacerbates this problem, wherein enormous volumes of plastic waste flow from high-income countries (HICs) to LMICs as a form of pollution and waste transfer [17]. Consequently, large volumes of waste remain uncollected, while the waste that is collected is often burnt in open spaces, which is an important contributor to global greenhouse gas (GHG) emissions [8]. It is estimated that the GHG emissions released from the burning of Coca-Cola Co’s plastic waste, alone, are equal to approximately three-quarters of the total GHG emissions generated from the firm’s entire global transport and distribution system [8]. In HICs, people with lower incomes and lower levels of educational attainment, as well as those who live in disadvantaged neighbourhoods, are more likely to consume a greater amount of SSBs than those better off [7, 18–23]. The same demographics have also been shown to be at higher risk of obesity, type 2 diabetes, and other non-communicable diseases associated with unhealthy diets [24, 25].

Although a considerable amount of public health work has looked at the impacts and drivers of the harms distributed via the global soft drink market, less public health attention has been devoted to examining wealth and income distribution in the same market. Wealth and income distribution, however, can impact health and social equity in several important ways. Key examples include the ways in which wealth and income distribution shape key structural determinants of health (e.g., wealth and income inequality; declining tax revenues that fund essential public services) [26–30], and the ways in which accumulated wealth is used to fund corporate strategies known to undermine public health and health equity [31]. Yet, corporations active in unhealthy commodity markets, like the soft drink market, often use arguments related to the ways in which they create and distribute wealth to highlight their economic ‘value’ and role in sustainable economic development, often as part of their efforts to challenge public policy intended to address the maldistribution of harms they perpetuate [32–35]. These economic arguments warrant analytical scrutiny from the public health community.

In most modern market economies, the market power of publicly listed corporations is a crucial determinant of wealth and income distribution, especially in the absence of robust government redistributive policies [36–38]. Market power, like all power constructs, is a contested subject [39]. Mainstream definitions of market power usually make reference to a firm’s ability to profitably raise prices above what would be possible in a competitive market environment [40, 41]. While this definition is useful in certain contexts, it does not consider that consumer prices are not always acutely relevant to the existence and use of power in markets (e.g., the power of Big Tech to control vast amounts of user data in digital platform markets) [42, 43]. Moreover, mainstream definitions of market power shed little light on the myriad other ways by which market power can negatively impact societal welfare beyond consumer price manipulation, such as the distributive impacts of foreign corporations that shift extracted wealth abroad, and the ability of powerful corporations to control working conditions and wages [43, 44]. More broadly construed definitions of market power are likely to be better placed to inform examinations into market power using a public health lens. Meagher (2020), for instance, considers market power to encompass the ability of a firm to shape market conditions [38]. This includes not only the ability to manipulate prices, but also the ability to shape the structure and governance of a market, to influence the path of innovation, to control the flows of information in a market, and to maximise the externalisation of costs [38]. Unlike mainstream definitions, this broader notion of market power takes into account its political impacts [45–47]. Firms with considerable market power can divert substantial wealth and resources to political activities, such as lobbying and campaign donations, which effectively reflect the purchasing of political power [45, 46]. Such power can then be used, for instance, to shape market governance or to protect a firm’s ability to externalise costs [38]. In concentrated industries, large firms are better positioned to coordinate their political efforts to influence industry-wide policy and regulation (i.e., policy and regulatory capture) [38, 48–50]. In addition, concentrated market power can also alter the balance of power between governments and corporate actors, which has the potential to manifest in increasing government hesitancy to implement policies and regulations that could threaten the profit-making abilities of dominant firms [51, 52].

The impact of market power on wealth and income inequality in any given commodity market depends on the relative distribution of wealth, income, and consumption (which reflects the source of revenue) [53]. Since the 1980s, an increasing proportion of global corporate wealth and income – much of which has been attributed to the extractive nature of market power in many economic sectors [36, 54, 55] – has been transferred to corporate shareholders and investors, a group over-represented by a small, wealthy elite mainly based in HICs [38, 56–61]. This phenomenon has often been described as the ‘shareholder primacy’ model of corporate governance [56]. In comparison, many consumer products, including many unhealthy commodities, are increasingly being consumed by citizens of LMICs, as well as lower socio-economic groups in HICs [15, 31, 48, 53, 62].

**Fig. 1:**
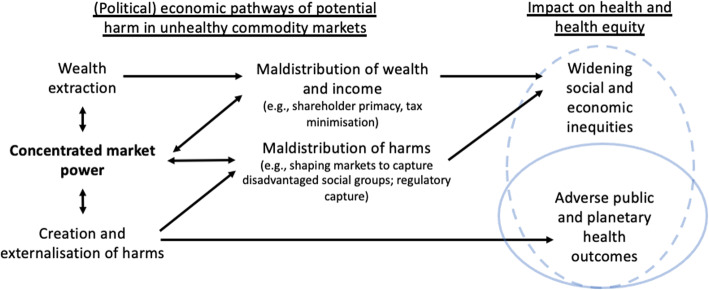
A broad overview of political economic pathways of potential harm in unhealthy commodity markets from a public health and health equity perspective

Compounding the distributive concerns of market power and shareholder primacy is that, over recent decades, corporations based in most jurisdictions around the world have increasingly been able to minimise their tax obligations [29, 63]. Traditionally, corporate income tax has been an important source of progressively levied government revenue, thereby playing an important role in funding essential public services and addressing socio-economic inequalities within societies [63–65]. A relative decline in government revenue from corporate income tax also shifts the tax burden onto other groups, including lower income households [30]. Moreover, in the face of rising corporate profits relative to gross domestic product (GDP) in many advanced economies, concomitant declines in relative income tax obligations effectively increase the ability of corporations to accumulate vast amounts of wealth, and thus, consolidate their market power [66]. Fundamentally, contemporary tax policy, market power and shareholder primacy are inextricably linked, together threatening distributive justice by underpinning a political economic system that allows, and even encourages, corporations to act for the benefit of the most, not least, advantaged members of society (see Fig. 1) [29, 67].

Taking the above into consideration, this paper aimed to explore how market power and corporate wealth and income distribution in the global soft drink market negatively impact public health and health equity. The particular aspects of corporate wealth and income distribution the paper explored were their distribution among corporate stakeholders (especially shareholders), as well as transfers to governments via corporate income tax payments. In doing so, the paper sought to contribute to a broad-based public health approach to market analysis, complementing other work that has examined the maldistribution of harms in unhealthy commodity markets. The findings were used to inform discussion of the appropriate role of the global soft drink market, and unhealthy commodity markets in general, as part of sustainable economic development.

## Methods

### Overview of methods

A range of dimensions and indicators were descriptively analysed to explore market power and corporate wealth and income distribution (see Table 1 for a summary of the indicators, levels of analysis and methods used) [68]. These dimensions are discussed in further detail in the following sections. Quantitative data were sourced from a range of business and market research databases.

**Table 1:** Dimensions and indicators used to explore market power and corporate wealth and income distribution

Dimension	Indicators	Level(s) of analysis	Description
**Market concentration (a source and outcome of market power)**	Herfindahl-Hirschman Index	Market (national)	The sum of the market shares of all active firms in a market which have been squared.
**Market power-mediated financial performance metrics**	Firm performance (market capitalization, earnings)	Firm and industry/sector	Descriptive analysis of company fundamental data.
**Allocation of wealth and resources to non-production practices**	Allocation of wealth and resources to practices related to pathways of harm	Firm and industry/sector	Descriptive analysis of selling, general and administration expenses, and wealth transfers to shareholders
**Corporate wealth and income distribution**	Distribution of consumption by geography	Firm and market	Quantitative analysis of sales revenue data
	Shareholder power and shareholder value ratios	Firm and industry	Quantitative analysis of data related to dividends, share repurchases, total revenues, and capital expenditures.
	Ownership structure and location	Firm and investor	Descriptive analysis of company equity ownership data and location of traded shares held.
	Effective tax rates	Firm and industry	Quantitative analysis of firm-level financial ratios

### Definition and categorisation of the global soft drink market

Euromonitor International (Passport) categorises the global soft drink market into the following product markets: carbonated soft drinks, juice, concentrates, sports drinks, energy drinks, ready to drink tea, ready to drink coffee, bottled water, and Asian specialty drinks [69].

In 2020, the size, by sales revenue, of the global soft drink market was US$772.5 billion, of which US$550.9 were ‘off-trade’ sales made through the following distribution channels: supermarkets, discounters, convenience stores, grocers, food and drink specialist stores, vending, home shopping, internet retailing and direct selling. The global soft drink market increased in size by 88% from 2006 to 2020. The carbonated soft drink market (US$273.0 billion total; US$171.9 billion ‘off-trade’) was the largest of the soft drink markets.

### Firm selection and overview

In 2020, Coca-Cola Co (20.8%) and PepsiCo (10.0%) held the largest market shares in the global soft drink market by a considerable margin. Their combined market share was greater than the combined market share of the next 78 firms (ranked by market share). As shown in Table 2, Coca-Cola Co (46.5%), and to a lesser extent PepsiCo (18.8%), held dominant positions in the global carbonated soft drink market – the largest of the soft drink product markets. Refer to supplementary file 1 for a brief overview of Coca-Cola Co and PepsiCo.

**Table 2:** Global and regional market shares held by Coca-Cola Co and PepsiCo, 2020

	Market share of carbonated soft drink market (excluding bottled water) (%)		Market share of total soft drink market* (%)	
	Coca-Cola Co	PepsiCo	Coca-Cola Co	PepsiCo
Asia Pacific	50.9	21.0	16.0	5.0
Australasia	57.2	14.8	29.3	8.4
Eastern Europe	40.3	19.6	16.6	12.4
Latin America	61.8	13.4	39.3	9.5
Middle East and Africa	29.5	23.2	14	10
North America	38.9	22.9	21.1	18.7
Western Europe	51.8	11.8	21.6	6.3
**World**	**46.5**	**18.8**	**20.8**	**10.0**

*Data source: Euromonitor International (Passport). Market share based on 2020 off-trade retails sales revenue*

*Product markets included, based on Passport’s categorisation of soft drink markets, are carbonated soft drinks; juice; ready-to-drink tea; energy drinks; ready-to-drink coffee; sports drinks; bottled water; concentrates; and Asian specialty drinks

### Market concentration

High market concentration, which occurs when only a limited number of firms control a market, has been described as both a symptom and cause of market power [70]. It is a symptom of market power in the sense that dominant firms often actively and successfully pursue strategies that increase market concentration (e.g., mergers and acquisitions, or through raising barriers to market entry), thereby creating a market environment conducive to generating sustained profits [71–73]. Concurrently, high market concentration acts as a source of market power by providing incumbent firms with structural and ‘competitive’ advantages relative to other market stakeholders, such as smaller rivals, new or potential market entrants, customers, consumers, suppliers, and employers [44, 55, 70, 74]. Market concentration is not a measure or quantification of market power per se. Market concentration analysis can, however, map the market structures that reflect the source and outcomes of the market power of incumbent firms.

As the product and geographic boundaries of markets need to be carefully defined in market concentration analysis [75, 76], our analysis focused specifically on carbonated soft drink markets at the national level. The carbonated soft drink market was chosen because it is the largest and arguably most important of the soft drink product markets from an economic perspective. Market concentration was calculated using the Herfindahl-Hirschman Index (HHI), a commonly used market concentration metric found by summing the square of the market share of every firm active in the respective market [77]. We drew from European Central Bank thresholds, as well as current and historical U.S. Department of Justice thresholds, in determining high (HHI > 1800) and very high (HHI > 2500) levels of concentration [78, 79]. A scatter plot was used to map market concentration levels (y-axis) against market size (x-axis). Data were sourced from Passport.

### Market power-mediated financial performance metrics

We analysed two financial performance metrics – market capitalisation and earnings – that are shaped by market power [80]. Our underlying assumption was that a considerable proportion of the market value and earnings of Coca-Cola Co and PepsiCo, like most modern publicly listed corporations, can be attributed to their market power [1, 36, 54, 55]. We analysed market capitalization values from 1962 to 2019 (based on available data). Market capitalization is a commonly used measure of the value of a company that is traded on a stock market, calculated by multiplying the total number of shares by the present share price [68]. It is typically understood to represent the expected future profits of a company, taking into account risks, and discounted to the present [80]. From a critical perspective, some scholars have argued that market capitalization can be more broadly understood as a ‘symbolic ritual’ that reflects the process of ‘dollarising’ the social, political and economic influence of corporations [80, 81]. We also explored earnings over the same period, using a commonly used indicator of corporate earnings – ‘Earnings before Interest, Tax, Depreciation and Amortisation’ (EBITDA) – that captures a firm’s earnings prior to financial and accounting deductions [82].

The financial performances of Coca-Cola Co and PepsiCo were compared to the average of the U.S. listed soft drink sector and the U.S. listed packaged food, meats, and soft drink sector. The Global Industry Classification Standard (GICS) was used for classification and data aggregation purposes. Company financial data were sourced from Standard and Poor’s Compustat (hereinafter Compustat) database [83]. All values were adjusted for inflation according to the 2010 U.S. Consumer Price Index [84].

### Allocation of wealth and resources to non-production practices

We analysed expenditure on certain non-production practices with the potential to undermine health and health equity. Specifically, we looked at advertising expenses, as well as selling, general, and administration expenses (encompassing other marketing expenses not disclosed by the firm in advertising expenses) from 1962 to 2019. There is substantial evidence in the public health literature outlining the role of extensive marketing of unhealthy products by dominant corporations in driving ill-health and health inequity [31, 48, 62, 85–88]. We also examined the wealth transferred to shareholders via dividends and share repurchases. As outlined in the introduction section, the disproportionate transfer of corporate wealth to shareholders relative to other stakeholders (e.g., workers) are an important driver of wealth and income inequalities, which are key structural determinants of health [26]. All values were adjusted for inflation according to the 2010 U.S. Consumer Price Index [84]. Data were sourced from Compustat.

### Corporate wealth and income distribution

Wealth and income distribution were explored by analysing a range of quantitative data from various databases. First, we examined the distribution of soft drink consumption by examining the annual off-trade soft drink revenue generated across available national markets from 2006 to 2020. These were aggregated by World Bank 2020 income level status. Data were sourced from Passport. Company share data were not available for more than half of these national markets; thus, we were unable to systematically analyse sales revenue generation by firm at the country level.

Second, we explored how Coca-Cola Co and PepsiCo distribute their wealth and income, focusing on transfers to shareholders and effective tax rates. We drew from an approach recommended by Hager and Baines (2020) to analyse shareholder power and shareholder value. The shareholder power ratio examines the combined value of dividends and share repurchases relative to capital expenditure. Capital expenditure is considered a proxy for the interests of ordinary workers by acting as a gauge for the relative commitment of firms to create jobs, innovate, and advance productivity through long term investment [64]. In comparison, the shareholder value ratio considers the total combined value of dividends and share repurchases relative to firm revenue [64]. Interpreted together, both ratios provide an indication of the proportion of wealth transfers to shareholders relative to other corporate stakeholders, including ‘ordinary’ workers. Both ratios were compared to the U.S.-listed soft drink sector average.

The ownership structures and investor locations of Coca-Cola Co and PepsiCo were also analysed. This involved identifying the largest shareholders in both firms. Previous studies have identified that a considerable proportion of the shares of the largest firms in many key sectors of the economy, including the food and drink sector, are held by only a handful of the world’s largest institutional investors [61, 89, 90]. This phenomenon, often captured by the term ‘common ownership’, has been described as a symptom of the increasing financialization of the global economy [56, 90, 91]. Several scholars have raised concerns about the role of ‘common ownership’ in reducing competition (thereby facilitating the concentration of market power) and driving publicly listed corporations to pursue the maximisation of shareholder value as their primary goal [56, 91, 92]. We also looked at the percentage of traded shares held according to the home country of the shareholder or investor. Ownership data were sourced from Orbis and Eikon databases [93, 94].

Finally, we described and compared the annual effective tax rates (total income tax relative to pre-tax income) of Coca-Cola Co and PepsiCo to sector medians. Data were sourced from Compustat. For the purposes of this analysis, the U.S. listed food and beverage sectors were classified using the Fama-French 49-industry classification system (‘Food’ and ‘Soda’) [95]. The period of analysis (1970–2020) was also determined by available data.

## Results

### Market concentration

**Fig. 2:**
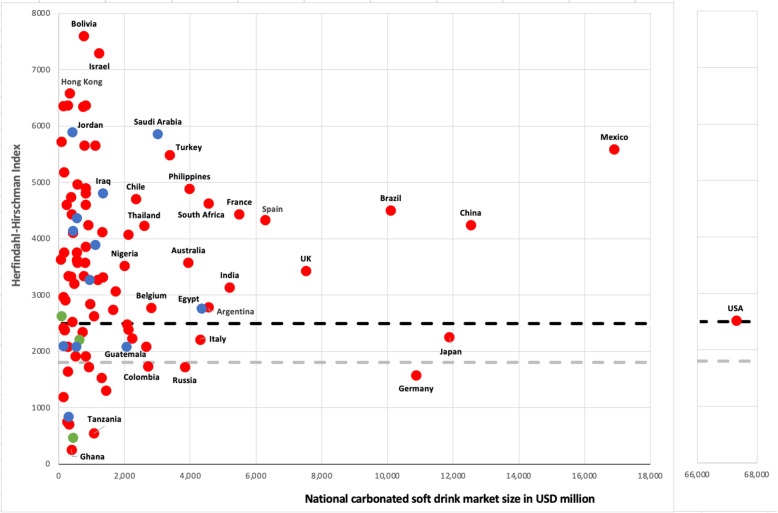
Scatter plot of market concentration (HHI) versus national carbonated soft drink market size, 2020. Data source: Euromonitor International (Passport). Red dots represent the markets in which Coca-Cola Co is the market leader. Blue dots are the markets in which PepsiCo is the market leader. Green dots are the markets in which neither firm is the market leader

The global carbonated soft drink market consists predominately of very highly concentrated markets (HHI > 2500; represented by the black dotted line in Fig. 2) at the national level. At the end of 2020, Coca-Cola Co was the market leader in most of the national markets analysed (*n* = 83/98) and held the second market position in the rest. PepsiCo was market leader in 12 markets, nine of which were in the Middle East. Only three national carbonated soft drink markets – Dominican Republic, Myanmar, and Angola – had a market leader other than Coca-Cola Co and PepsiCo.

### Market power-mediated financial performance metrics: market capitalization and EBITDA

**Fig. 3:**
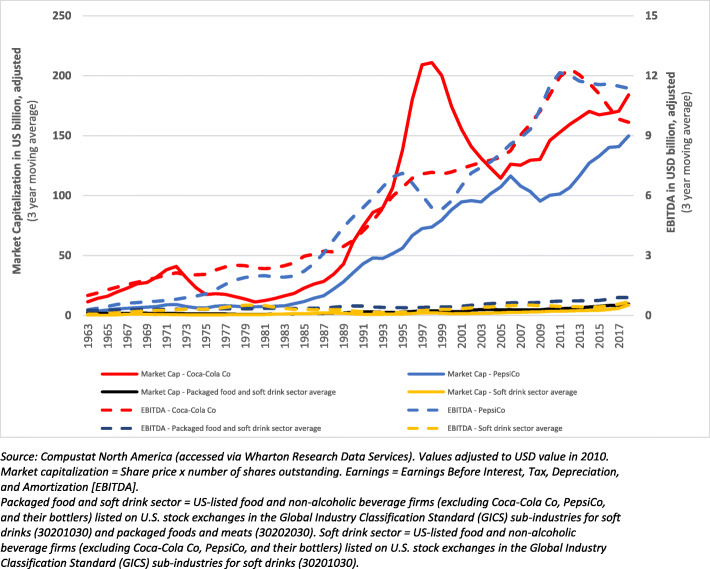
Market capitalization and earnings (adjusted to 2010 USD value) of Coca-Cola Co and PepsiCo relative to the sector averages, 1962–2019

As of May 2021, Coca-Cola Co had a market capitalization of US$231.3 billion, making it the world’s 37th largest publicly listed corporation, and second largest food and beverage corporation behind Nestlé [96]. PepsiCo, with a market capitalization of US$199.2 billion, was the world’s 50th largest publicly listed corporation and third largest food and beverage corporation [96]. Adjusted for inflation, the market capitalization values of Coca-Cola Co and PepsiCo were seen to be consistently much greater than the U.S. listed packaged food and soft drink sectors over the period 1962 to 2019 (see Fig. 3).

The earnings of both Coca-Cola Co and PepsiCo have also been considerably greater than the sector average over the same period (see Fig. 3). Over the 50-year period between 1970 and 2019, Coca-Cola Co accumulated a total of US$276.3 billion, and PepsiCo a total of US$268.7 billion, more than the U.S. sector average (adjusted for inflation; 2010 USD values).

### Allocation of wealth and resources to non-production practices

**Fig. 4:**
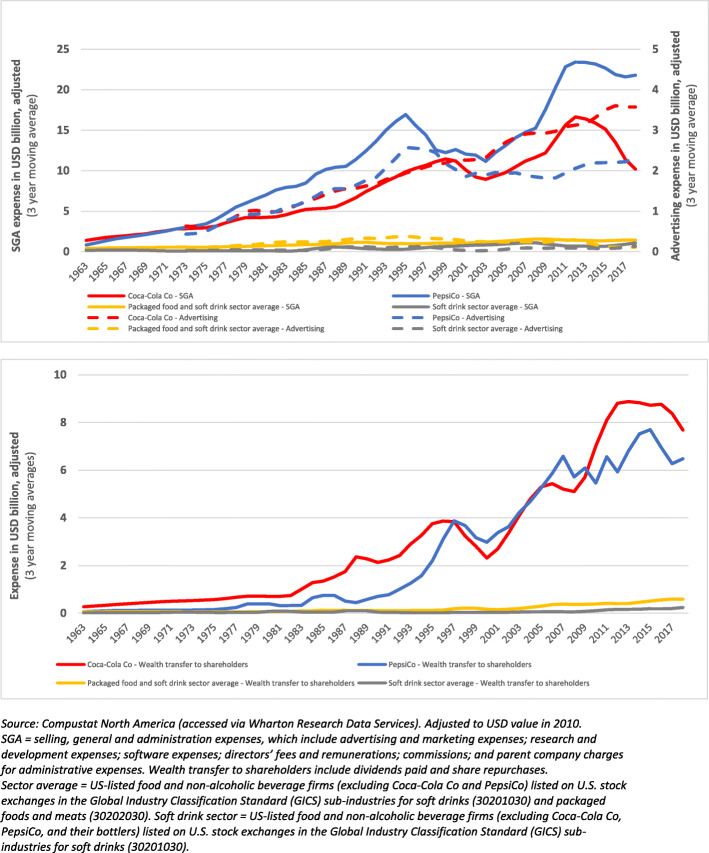
Allocation of funds (adjusted to 2010 USD values) to advertising and shareholder wealth transfer practices by Coca-Cola Co and PepsiCo relative to sector averages 1962–2019

Coca-Cola Co and PepsiCo allocate a substantial amount of funds to advertising; selling, general and administration (SGA) practices (which encompasses advertising and other marketing related expenses); and wealth transfers to shareholders via dividends and share repurchases (Fig. 4). Adjusted to 2010 USD values, over the 40-year period between 1980 and 2019, Coca-Cola disclosed US$385.7 billion on SGA practices (8.4 times greater than the U.S. listed packaged food and soft drink sector average) and US$90.5 billion on advertising (8.9 times the sector average). Over the same period, PepsiCo disclosed US$584.3 billion on SGA practices (12.7 times the sector average) and US$74.9 billion on advertising (7.4 times the sector average). Since the 1980s, the amount of wealth that both firms have transferred to shareholders via dividends and share repurchases has also increased considerably in absolute terms and relative to the sector average. Adjusted to 2010 USD values, between 1980 and 2019, Coca-Cola Co and PepsiCo allocated US$170.3 billion (17 times the sector average) and US$ 141.1 billion (14.1 times the sector average), respectively, to these practices.

### Corporate wealth and income distribution

#### Distribution of consumption by geography

**Fig. 5:**
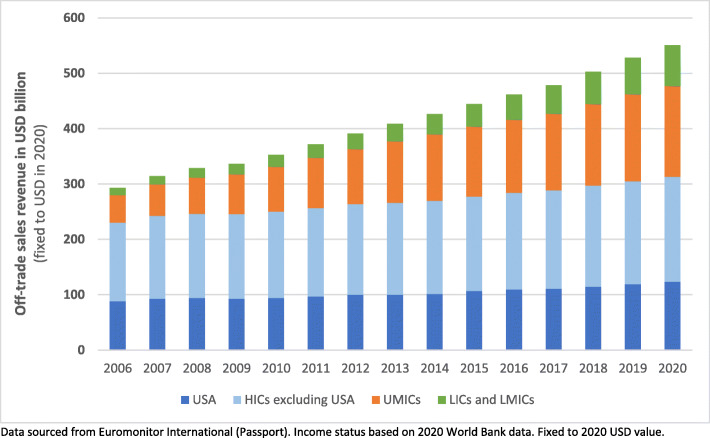
*Revenue generated from soft drink markets, by World Bank income status, 2006–2020*

An increasing proportion of carbonated soft drink revenue is generated from consumers in low-income countries (LICs), lower middle-income countries (lower MICs) and upper middle-income countries (upper MICs), relative to consumers in HICs (refer to Fig. 5). From 2006 to 2020, the total revenue (fixed to 2020 USD value) generated in LIC markets increased by 816% (0.46 to 3.78 billion USD); in lower MIC markets by 403% (5.68 to 22.93 billion USD); in upper MIC markets by 250% (20.74% to 51.82 billion USD); and in HIC countries by 21% (77.23 to 99.40 billion USD).

### Shareholder power and shareholder value

**Fig. 6:**
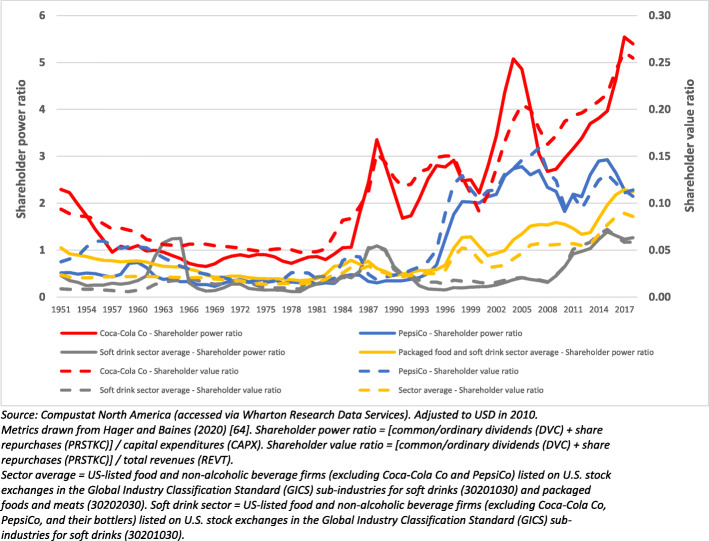
Shareholder power and value ratios of Coca-Cola Co and PepsiCo relative to sector averages, 1950–2020

Since the 1980s, an increasing proportion of wealth has been distributed by both firms to their shareholders relative to capital expenditures (shareholder power ratio) and total revenue (shareholder value ratio) (Fig. 6). Notably, in 2017, the shareholder power ratio of Coca-Cola Co was six times greater than what it was in 1980, and the shareholder value ratio more than 5 times greater than in 1980. This demonstrates that, over time, an increasing proportion of the wealth generated by Coca-Cola Co, and to a lesser extent PepsiCo, has been transferred to shareholders at the expense of other stakeholders, including employees. It can also be seen that the shareholder power and value ratios of Coca-Cola Co, and to a lesser extent PepsiCo, have generally been much greater than sector averages.

#### Ownership structure and shareholder location

The majority of shares in both Coca-Cola Co and PepsiCo are held by institutional investors, encompassing mutual and pension funds, other financial institutions, banks, and insurance firms. As of 2020, Coca-Cola Co’s top 10 investors, all of which were US-based, held 37.2% of the firm’s equity stakes, combined. These institutional investors included Berkshire Hathaway (9.3%), Vanguard Group (7.5%), Blackrock (6.9%), and State Street (4.4%). Nine of the top 10 investors in PepsiCo were US-based (Norway’s Sovereign Wealth Fund was the exception), which in combination held 31% of the firm’s equity stakes. These investors included Vanguard Group (9.0%), Blackrock (7.5%), State Street (4.8%), and Bank of America (1.9%).

As of June 2021, a large majority of traded shares of both firms were held by investors and shareholders based in the U. S, with almost all traded shares held by investors and shareholders in HICs (see Table 3). None of the traded shares were held by investors and shareholders based in LICs, with only a very small percentage held by investors and shareholders based in lower MICs and upper MICs.

**Table 3:** Investor location, by World bank income level, of traded shares of Coca-Cola Co and PepsiCo

	HICs			UMICs	LMICs	LICs
	U.S.	Western Europe	*All HICs*			
Coca-Cola Co	82.27%	13.44%	*99.95%*	0.04435%	0.005094%	0%
PepsiCo	76.55%	17.14%	*99.96%*	0.03454%	0.008756%	0%

Data sourced from Eikon database, based on 30 June 2021 filings. Income status based on 2020 World Bank data. HICs = high-income countries; UMICs = upper middle-income countries; LMICs = lower middle-income countries; LICs = low-income countries.

#### Corporate effective tax rates

**Fig. 7:**
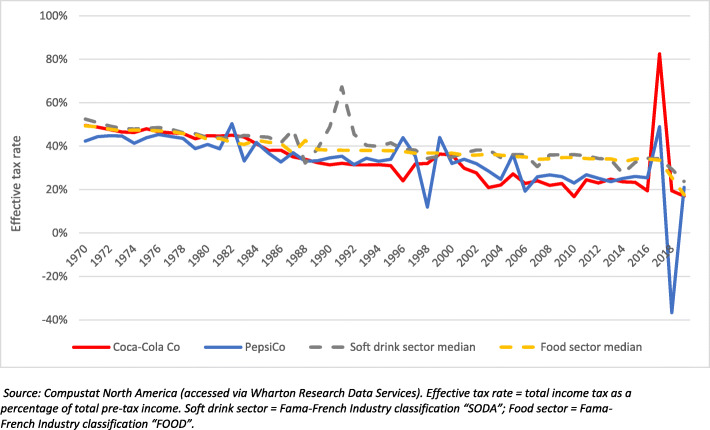
The annual effective tax rates of Coca-Cola Co and PepsiCo relative to sector medians, 1970 to 2019

Over the period 1970 to 2016, the effective tax rates of Coca-Cola Co and PepsiCo declined from 50 and 42%, respectively, to 19 and 25% (Fig. 7). From 1980 onwards, the effective tax rates of both Coca-Cola Co and PepsiCo were typically below the U.S. listed food and soft drink sector medians. Findings from 2017 onwards were likely affected by U.S. corporate statutory income tax rate changes, which went from 35 to 21% in 2018 [97]. In 2017, Coca-Cola Co had an effective tax rate of 82% and PepsiCo of 49%, with these high numbers likely representing financial restructuring in anticipation of the upcoming tax changes. In 2018 and 2019, Coca-Cola Co had annual effective tax rates of 19 and 17%, respectively, and PepsiCo had effective tax rates of − 37% (signifying that PepsiCo received a net tax rebate) and 21%, respectively.

## Discussion

This paper showed that the global carbonated soft drink market – the largest of the soft drink product markets – predominately consists of highly concentrated markets at the national level. These are mostly dominated by Coca-Cola Co, and to a lesser extent, PepsiCo. This patchwork of market concentration likely reflects the considerable market power of these global corporations, illustrating the extent to which both firms have managed to spread, penetrate, and shape a large number of markets around the world [44, 55, 70, 72–74].

Both Coca-Cola Co and PepsiCo have been valued at, and have generated, earnings at levels considerably greater than the average U.S.-listed packaged food and non-alcoholic beverage company over a sustained period. This is likely to be, in part, attributable to their extensive market power [36, 54]. Both firms clearly have the ability to divert substantial wealth and resources towards non-production practices, some of which have the potential to undermine public health and health equity. Our findings, for instance, highlight how Coca-Cola Co and PepsiCo have allocated billions of USD every year, for decades, to advertise their products, and likely also allocate substantial funds to other sophisticated forms of marketing (such as public relation campaigns and sport sponsorships) not covered under ‘advertising’ in their corporate reporting [98, 99]. Such enormous marketing budgets aim to create, maintain and increase consumer demand around intangible benefits (e.g. enjoyment, happiness, social status) [1]. In doing so, they likely drive the overall production and consumption-related burden of health and ecological harms externalised by the market [62, 100–102]. From a health equity perspective, evidence suggests that the marketing of soft drink products, as with many unhealthy commodities, is also increasingly being directed at disadvantaged groups, including children, adolescents, minority groups, people living in disadvantaged neighbourhoods, and, more broadly, consumers in LMICs [31, 98, 102–105]. These population groups tend to be more vulnerable to industry marketing tactics, and are likely to be more constrained in exercising choice related to consumption [31].

The market power of Coca-Cola Co and PepsiCo also likely acts as an important source of their political influence, an issue well described in the public health literature [1]. Both firms, for instance, spend many millions in USD on corporate political practices, such as lobbying and political contributions, across a number of jurisdictions [106, 107]. In many cases, these practices form part of a broader strategy to block or delay governments from regulating their products and practices [49, 108–110]. The size of Coca-Cola Co and PepsiCo, and the concentrated nature of their key markets, also likely provides both firms with a structural form of political power relative to governments [51, 52]. Like other large and powerful corporations active in unhealthy commodity industries, dominant soft drink corporations have been known to refer to their extensive market and economic power – e.g., the number of workers they employ, the investment opportunities they provide, and the tax revenues they generate – to argue that government policies and regulations designed to address the harms they externalise could adversely impact the national economy [111–114]. Relatedly, funding disclosures made by Coca-Cola Co over the past decade reveal how the firm has had the financial means to contribute a substantial amount of money – nearly 150 million USD in total between 2010 and 2019 – to a number of academic, research and other institutes and organisations [115, 116]. Between 2010 and 2019, for example, Coca-Cola Co gave more than US$10 million to three research institutes and collaborations – ISCOLE, the International Life Sciences Institute, and the Global Energy Balance Network – that have all been criticised for attempting to shift the blame of diet-related chronic disease away from Coca-Cola Co’s products and operations [1, 117, 118]. The formation of these relationships likely plays an important role in increasing Coca-Cola’s ability to shape the way in which its health-harming products are regulated [99, 119–121].

### The ‘double burden of maldistribution’ in unhealthy commodity markets

Consistent with the ‘shareholder primacy’ model of corporate governance, our paper has demonstrated how, in recent decades, both Coca-Cola Co and PepsiCo have transferred an increasing proportion of their wealth and income to their shareholders and investors, most of which are based in HICs, via dividends and share repurchases. For Coca-Cola Co, and to a lesser extent PepsiCo, this proportion has been considerably greater than the sector average over a sustained period of time, supporting the notion that market power has played an important role in maximising wealth for the shareholders of these corporations [54].

In comparison, our findings highlight how the distribution of soft drink consumption is increasingly being skewed towards consumers in LMICs. Although our analysis only looked at cross-border wealth transfers, evidence suggests that lower socio-economic groups, a group over-represented by non-shareholders and holders of only limited amounts of corporate equity, consume greater amounts of soft drink products compared to higher socio-economic groups [7, 18–23, 53]. Thus, disadvantaged social groups are an increasingly important source of wealth for the shareholders of the dominant soft drink corporations. This is a trend inextricably linked to the same social groups also facing an increasingly disproportionate burden of consumption-related harms externalised by the market.

Compounding the distributive impacts of corporate wealth and income distribution to shareholders and the distribution of consumption is that, over the last 50 years, the proportion of wealth redistributed by PepsiCo and Coca-Cola Co to the public through income tax revenues has decreased substantially. Our findings revealed that the annual effective tax rates of both firms have declined to around a half of the levels seen in the late-1960s, signifying that over the past 50 years the proportion of the wealth redistributed to the public in the form of income tax revenues from these companies has decreased substantially. Thus, it can be understood that Coca-Cola and PepsiCo have been transferring a smaller proportion of their generated wealth to governments that, concomitantly, have had to spend increasing amounts to cover the health, social and ecological-related costs related to their business operations and activities [2, 122]. It is worth noting that both firms are subject to other types of taxes (e.g. sales, use, excise, value-added and payroll) not covered in the analysis, although, in general, these tend to be substantially less than income tax payments [123].

The downward trend in the effective tax rates of Coca-Cola Co and PepsiCo has likely been facilitated by the gradually declining statutory corporate tax rates across many countries in recent decades, a phenomenon resulting from, at least in part, the successful lobbying of global corporations in recent decades [29, 30, 97]. In addition, global corporations like Coca-Cola and PepsiCo have become particularly savvy at structuring their organisations and activities to reduce their tax obligations. This includes the technique of transfer pricing, involving the pricing of transactions between firms owned or controlled by the corporate entity in order to take advantage of jurisdictions with lower tax rates [124]. While corporate efforts to minimise tax are typically within the law, there are several instances of soft drink corporations acting illegally reduce their taxes. In 2020, for instance, the US Tax Court judged that Coca-Cola Co had illegally transferred its profits to low-tax jurisdictions between 2007 and 2009 to avoid about US$9 billion in income tax obligations [125]. Both firms also continue to receive large public subsidies, including in the form of tax deductions, to deploy a number of their corporate strategies that have the potential to undermine public health, such as marketing and making ‘charitable’ contributions to eligible organisations (e.g. eligible university foundations) [126, 127]. The ongoing use of public money by corporations to deploy strategies that could undermine the health of the public is problematic and unsustainable [128].

### Towards just and sustainable economic development

Euromonitor, the global market research company, predicts that the global soft drink market will increase in size in the coming years, with most of the growth expected to occur in LMICs. It has been forecast that, in 2024, global sales volume and revenue figures will be 5.0 and 16.1% greater, respectively, than what they were in 2019 [129].

Given the market’s maldistributive impacts, it is necessary to question the appropriate role of the global soft drink market as part of the current sustainability agendas of high-level global institutions and processes. Indeed, if the status quo is maintained, it is difficult to envision how the expansion of the global soft drink market can be made compatible with the pursuit of achieving a number of the United Nation’s (UN) Sustainable Development Goals, such as ensuring healthy lives and promoting well-being for all at all ages; ending all forms of malnutrition; reducing inequality within and among countries; ensuring sustainable consumption and production patterns; conserving the oceans, seas and marine resources for sustainable development; and protecting, restoring and promoting sustainable use of terrestrial ecosystems [130]. Moreover, the increasing role of powerful corporations active in unhealthy commodity markets, including the soft drink market, in influencing high-level agendas, such as the UN Food Systems Summit, presents a substantial conflict between corporate and public health interests [131, 132]. In many cases, these corporations position themselves as ‘part of the solution’ to the very problems they play a key role in creating and perpetuating [35].

### The role of the investment community

This paper has demonstrated the extent to which the shares of Coca-Cola Co and PepsiCo are held by large institutional investors, a finding consistent with the rise of ‘common ownership’ across many key sectors of the global economy [90, 133]. This raises the question about the extent to which institutional investment could be used as a lever, largely through shaping corporate governance, to drive substantial change in the global soft drink market. At least in principle, there are a range of strategies that could be used by the investment community to drive Coca-Cola Co and PepsiCo to pursue change for the betterment of society and the environment, including positive screening, divestment, and engagement [133]. Encouragingly, at least from a public health perspective, activist investors are already pushing for Coca-Cola Co and PepsiCo to be more transparent about the impact of their soft drink products on public health [134]. Having said that, though, in 2020, only 7% of Coca-Cola’s shareholders and 11% of PepsiCo’s shareholders voted in favour of this specific shareholder resolution [134]. At least for the moment, shareholder and investor appetite for corporate actions that might jeopardise short-term financial gains appears to be rather limited. This is an argument further supported by the  recent dismissal of Danone’s CEO for allegedly pursuing ‘non-financial’ goals, such as sustainability, to the perceived detriment of the corporation’s short-term financial performance [135].

### Strengths, limitations, and suggestions for future work

A key strength of this paper is that it sourced and analysed data from a diverse range of company, market and industry databases that are not often used, and seldom integrated, in public health research. The databases used to source data for our analysis, as well as other business databases, have the potential to play an important role in strengthening research, such as that in the field of the corporate and commercial determinants of health, that attempts to understand, identify, and monitor the impacts of business on health.

This paper has several important limitations. Firstly, the paper focused solely on some of the potential negative impacts of the global soft drink market, and did not consider potential positive impacts. We also recognise that our approach to market analysis is not comprehensive, as it did not consider aspects such as wages and working conditions. Nevertheless, the kind of analysis presented in this paper is likely to serve as a useful point of departure for the development of a broad-based interdisciplinary research program aimed at comprehensively assessing the ways in businesses and markets influence public health and health equity. Future work could, for instance, incorporate assessments of how markets contribute to social equity, respect human rights, provide social needs, promote the development of innovations that provide real social benefits, encourage human creativity and freedoms, and influence and co-evolve with the socio-political institutions within which they are embedded [68, 136–139]. Such work could also incorporate planetary health outcomes, such as impacts on ecological systems and processes, and impact on animal welfare [136, 140].

While our analysis put the spotlight on large and powerful soft drink corporations, the paper also calls into question the underlying legal, regulatory, and institutional frameworks that allow, promote and perpetuate a lack of corporate accountability to society and the environment, the concentration of market power, and the unjust distribution of wealth and income. In this respect, future research could explore government levers that have the potential to protect and promote public and planetary health via addressing issues such as market concentration, market power and shareholder primacy. These may include relatively short-term remedies, such as ensuring that public money does not subsidise corporate practices that undermine health (e.g., tax deductions for marketing unhealthy commodities). More broadly, and given the increasing social and political momentum behind current anti-monopoly, inclusive/stakeholder capitalism, and economic de-growth movements [38, 138, 141–144], a potential avenue for future work could be to examine the plausibility and feasibility of integrating public health interests and values into future antitrust and corporate law and policy reforms.

## Conclusion

Market power and corporate wealth and income distribution in the global soft drink market likely compound the market’s maldistribution of harms, as well as indirectly influence health by contributing to a range of social and economic inequalities. Indeed, a ‘double burden of maldistribution’ pattern can be seen, wherein the wealth of the shareholders of the market’s dominant corporations, a group over-represented by a small and wealthy elite, is maximised largely at the expense of the welfare of the lower socioeconomic classes of HICs, the citizens and governments of LMICs, the environment, and indeed, future generations.

Marked transformation will surely be needed if the global soft drink market is to play a role in sustainable economic development. Fundamentally, the persistence and perpetuation of these concerns related to health inequity and distributive justice can be understood as systemic and structural features of modern capitalism. As such, industrial and market transformation – and more broadly, the move towards a socially just and sustainable future – will likely only be achieved through the transformation of the underlying legal, regulatory, and institutional frameworks that have become characteristic of the modern capitalist era.

## Supplementary Information

**Additional file 1..** Supplementary file 1: A brief overview of the Coca-Cola Company and PepsiCo

## Data Availability

Not applicable.
