# Supplementary material for: Maximising the wealth of few at the expense of the health of many: a public health analysis of market power and corporate wealth and income distribution in the global soft drink market
Source: Global Health. 2021 Dec 2;17:138. doi: 10.1186/s12992-021-00781-6 (PMC8641192; doi:10.1186/s12992-021-00781-6)
Supplement: Supplementary file 1 — Additional file 1.. Supplementary file 1: A brief overview of the Coca-Cola Company and PepsiCo [file 12992_2021_781_MOESM1_ESM.docx]

**Supplementary file 1: A brief overview of the Coca-Cola Company and PepsiCo**

*The Coca-Cola Company*

The first non-alcoholic Coca-Cola product was created in 1886 in Georgia, USA [1,2]. Coca-Cola Co was first incorporated in 1892, and as early as 1906 had begun to globalise its production, with bottling operations beginning in Canada, Cuba and Panama [3,4]. By the time World War 2 had started, Coca-Cola was being bottled in 44 countries. By 1959, this number increased to over 100, and as of 2020, the firm’s products were ‘officially’ sold in every single country and territory bar North Korea and Cuba [4,5].

The so-called ‘Coca-Cola System’ of controlling (but not owning) bottling operations is long-standing and unique within the industry [4,6]. Rather than directly owning most of the companies that bottle Coca-Cola products around the world, Coca-Cola Co instead relies upon having large minority equity stakes to exert influence over the corporate governance of many of its key bottlers. Importantly, this ownership structure means that Coca-Cola Co does not have to take full responsibility for bottling operations and associated financial risks [7].

*PepsiCo*

In 1898, the soft drink Pepsi was created in North Carolina, USA [8]. The Pepsi-Cola Company was first incorporated in 1919 in Delaware, USA [9]. From its outset, the Pepsi-Cola Company has largely operated in the shadow of its more popular and economically powerful rival, Coca-Cola Co. This likely played a key factor in Pepsi-Cola’s decision to merge with Frito-Lay in 1965, forming PepsiCo, leading to the company’s diversification into snack foods and other food products [10]. In 2019, 46% of PepsiCo’s total sales revenue was generated from soft drink sales, with the remainder generated from food sales [11]. As of 2020, PepsiCo’s products were sold in more than 200 countries and territories around the world [10].

**References**

1. Adams, W.L. Is this the Real Thing? Coca-Cola’s Secret Formula ‘Discovered’. Availabe online: <https://newsfeed.time.com/2011/02/15/is-this-the-real-thing-coca-colas-secret-formula-discovered/> (accessed on 5 January 2021).

2. Lemelson-MIT. John Pemberton. Availabe online: <https://lemelson.mit.edu/resources/john-pemberton> (accessed on 5 January 2021).

3. Georgia Historical Society. Marker Monday: The Birthplace of Coca-Cola. Availabe online: <https://georgiahistory.com/marker-monday-the-birthplace-of-coca-cola/> (accessed on 5 January 2021).

4. The Coca-Cola Company. *125 years of sharing happiness*; The Coca-Cola Company: Atlanta, Georga, 2011.

5. Kershner, E. Countries that Surprisingly Don’t Sell Coca-Cola. Availabe online: <https://www.worldatlas.com/articles/countries-that-surprisingly-don-t-sell-coca-cola.html#:~:text=There%20are%20only%20two%20countries,trade%20sanctions%20on%20North%20Korea>. (accessed on 5 January 2021).

6. The Coca-Cola Company. The Coca-Cola System. Availabe online: <https://www.coca-colacompany.com/company/coca-cola-system#:~:text=COCA%2DCOLA%20SYSTEM-,THE%20COCA%2DCOLA%20SYSTEM,community%20where%20we%20do%20business.&text=The%20primary%20way%20that%20our,and%20syrups%20to%20bottling%20operations> (accessed on 4 December 2020).

7. Financial Times. Pepsi bottles it. Availabe online: <https://www.ft.com/content/6cc01f6e-2d83-11de-9eba-00144feabdc0> (accessed on

8. Bellis, M. The History of Pepsi Cola. Availabe online: <https://www.thoughtco.com/history-of-pepsi-cola-1991656> (accessed on 5 January 2021).

9. Leonard, C. Caleb Bradham’s Soda Fountain Flavour “Brad’s Drink” Became Pepsi-Cola Today in 1898. Availabe online: <https://nowweknowem.wordpress.com/2013/08/28/caleb-bradhams-soda-fountain-flavor-brads-drink-became-pepsi-cola-today-in-1898-now-we-know-em/> (accessed on 6 January 2021).

10. PepsiCo. About the Company. Availabe online: <https://www.pepsico.com/about/about-the-company> (accessed on 6 January 2021).

11. PepsiCo. *Annual Report 2019*; PepsiCo: 2019.
